# Molecular portraits and trastuzumab responsiveness of estrogen receptor-positive, progesterone receptor-positive, and HER2-positive breast cancer

**DOI:** 10.7150/thno.35730

**Published:** 2019-07-09

**Authors:** Shen Zhao, Xi-Yu Liu, Xi Jin, Ding Ma, Yi Xiao, Zhi-Ming Shao, Yi-Zhou Jiang

**Affiliations:** Department of Breast Surgery, Fudan University Shanghai Cancer Center; Cancer Institute, Fudan University Shanghai Cancer Center; Department of Oncology, Shanghai Medical College, Fudan University, P.R. China

**Keywords:** triple-positive breast cancer, multiomics, molecular classification, luminal A, trastuzumab

## Abstract

**Background**: Estrogen receptor-positive, progesterone receptor-positive, and HER2-positive breast cancers (triple-positive breast cancers, TPBCs) account for 5% to 10% of all breast cancers. The clinical and molecular features of TPBCs remain elusive. In this study, we aim to analyze the multiomics landscape and responsiveness of TPBCs to trastuzumab.

**Methods**: We employed five cohorts. The first cohort was from the Surveillance, Epidemiology, and End Results database (n=32,056) and was used to determine the clinical characteristics of TPBC. The second, third and fourth cohorts were from The Cancer Genome Atlas (n=162), GSE2603 (n=37) and GSE2109 (n=30) datasets, respectively, and were used to examine the genomic features and molecular classification of TPBC. The fifth cohort comprised TPBC patients treated at Fudan University Shanghai Cancer Center (FUSCC, n=171) and was used to investigate an immunohistochemistry-defined luminal A-like subgroup of TPBC.

**Results**: Patients with TPBC had a significantly better prognosis than those with ER-PR-HER2+ breast cancer. Genomic analysis revealed that TPBCs showed a lower *TP53* mutation rate (30% vs. 69%, *P* < 0.001) and lower levels of HER2 mRNA and protein expression than ER-PR-HER2+ breast cancers. More than 40% of TPBCs were classified as the luminal A intrinsic subtype, with an even lower HER2 expression level. Based on the immunohistochemical detection of CDCA8, BCL2 and STC2, we identified a luminal A-like subgroup of TPBCs in the FUSCC cohort (CDCA8-negative, BCL2- and/or STC2-positive). Patients with luminal A-like TPBC had a better prognosis and benefited less from trastuzumab than those with TPBC of other subtypes.

**Conclusions**: TPBCs consist of clinically and genomically heterogeneous subgroups that may require different therapeutic strategies. The luminal A-like subgroup of TPBCs is associated with a better prognosis and reduced benefit from trastuzumab.

## Introduction

Breast cancer is increasingly recognized as a heterogeneous disease that exhibits substantial differences in terms of pathological features, biological behavior and gene expression profiles. It is widely accepted that breast cancers can be classified into four molecular subtypes (luminal A, luminal B, HER2-enriched and basal-like) according to the gene expression patterns 1, 2. In clinical practice, a simplified classification based on the detection of estrogen receptor (ER), progesterone receptor (PR), human epidermal growth factor receptor 2 (HER2) and Ki-67 by immunohistochemistry (IHC) and/or fluorescence in situ hybridization has been adopted as a substitute 3. The classification results help guide treatment decisions.

Approximately 15-20% of breast cancers overexpress HER2, and nearly half of these HER2+ breast cancers also express hormone receptors (HRs) 4, 5. Previous retrospective studies reported a better prognosis for HR+HER2+ breast cancers than for HR-HER2+ breast cancers 6. However, few studies have focused specifically on the clinical features and prognosis of ER+PR+HER2+ breast cancers (triple-positive breast cancers, TPBCs). As for treatment, although HER2-targeted therapy (e.g., trastuzumab) can improve the prognosis of patients with HER2+ breast cancer regardless of HR status, the benefit may be lower in HR+ patients than in HR- patients 7.

Compared with the surrogate IHC-based classification, the intrinsic molecular classification based on gene expression profiling might better reveal the molecular essence and indicate treatment sensitivity 8-10. Although TPBC was all categorized as the luminal B subtype according to the surrogate IHC classification 3, it is heterogeneous according to the intrinsic molecular classification. Previous studies have demonstrated that HR+HER2+ breast cancers comprised at least luminal A, luminal B and HER2-enriched intrinsic subtypes, and the intrinsic molecular classification was associated with the tumor sensitivity to HER2-targeted therapy 11, 12. Certain subgroups of TPBCs, such as luminal A, may not be sensitive to HER2-targeted therapy.

In this study, we employed five well-characterized cohorts of breast cancer patients to comprehensively study the clinicopathologic and molecular features of TPBCs. In addition, we developed and validated a clinically practical method based on IHC to identify a luminal A-like subgroup of TPBCs, which may benefit less from trastuzumab.

## Methods

### Study Cohorts

Our study comprised five cohorts. The first cohort included 32,056 patients with HER2-positive breast cancer identified from the Surveillance, Epidemiology and End Results (SEER) database. We used this cohort to examine the clinicopathologic features and prognoses of HER2-positive breast cancers according to ER and PR status. The second cohort comprised 162 patients with HER2-positive breast cancer identified from The Cancer Genome Atlas (TCGA). Based on the somatic mutation, copy number, RNA-seq and reverse-phase protein array (rppa) protein expression data, we unraveled the genomic landscape and examined the HER2 expression level of HER2-positive breast cancers according to ER and PR status. The third and fourth cohorts were from two publicly available microarray datasets (GSE2603 and GSE2109), which included 37 and 30 patients with TPBC, respectively. We used the TCGA cohort and these two cohorts to study the intrinsic molecular classification of TPBCs and to select the genes that can be used to identify luminal A subtype TPBCs. The fifth cohort was a prospective observational cohort, which included 171 consecutive TPBC patients treated at Fudan University Shanghai Cancer Center (FUSCC) between 2007 and 2014.

Detailed information about our study cohort, bioinformatics and immunohistochemical analysis methods can be found in the Supplementary File.

### Statistical analysis

Student's t test was used to compare differences in continuous variables, while Pearson's chi-square test and Fisher's exact test were used to compare differences in categorical variables. For multiple testing adjustment, a false discovery rate was calculated using the R function “p.adjust”. In the SEER and TCGA cohorts, breast cancer-specific survival (BCSS) was defined as the interval from diagnosis to death due to breast cancer. Overall survival (OS) was defined as the interval from diagnosis to death from any cause. Disease-free survival (DFS) was defined as the interval from diagnosis to the first recurrence or death. In the FUSCC cohort, relapse-free survival (RFS) was defined as the time from diagnosis to the first relapse, incidence of contralateral breast cancer, or death from any cause. The survival curves were plotted using the Kaplan-Meier method, and the survival differences between groups were compared using the log-rank test. Multivariate survival analyses were performed using the Cox proportional hazards model, and the results were reported as hazard ratios (HRs) with 95% confidence intervals (CIs).

Statistical analyses were performed using R version 3.5.3 (R Foundation for Statistical Computing, Vienna, Austria). All tests were two-sided, and a *P* value < 0.05 was considered statistically significant.

## Results

### Clinicopathologic features and prognosis of triple-positive breast cancers

We outlined the demographic, tumor, and treatment characteristics of patients with HER2-positive breast cancers in the SEER cohort according to ER and PR status (Table S1). TPBCs accounted for 50.8% of HER2-positive breast cancers, while ER+PR-HER2+, ER-PR+HER2+ and ER-PR-HER2+ breast cancers accounted for 18.1%, 1.9% and 29.2%, respectively. Compared with ER-PR-HER2+ patients (≥ 50 years, 69.1%), TPBC (≥ 50 years, 62.8%) or ER-PR+HER2+ patients (≥ 50 years, 64.2%) tended to be younger, while ER+PR-HER2+ patients (≥ 50 years, 74.7%) tended to be older. Tumors of TPBC and ER+PR-HER2+ breast cancer were less frequently of a higher grade (grade 3 or undifferentiated, 48.9% and 58.6%, respectively) than those of ER-PR-HER2+ breast cancer (grade 3 or undifferentiated, 75.5%). The tumor size of TPBC and ER+PR-HER2+ breast cancer was relatively smaller (T1, 52.8% and 51.4%, respectively) than that of ER-PR-HER2+ breast cancer (T1, 46.6%). Patients with TPBC or ER+PR-HER2+ breast cancer were less likely to be diagnosed with node-positive disease (38.4% and 38.1%, respectively) than those with ER-PR-HER2+ breast cancer (42.3%). In addition, the rate of lumpectomy in patients with TPBC or ER+PR-HER2+ breast cancer was higher (51.2% and 46.9%) than that in patients with ER-PR-HER2+ breast cancer (42.7%).

**Figure 1:**
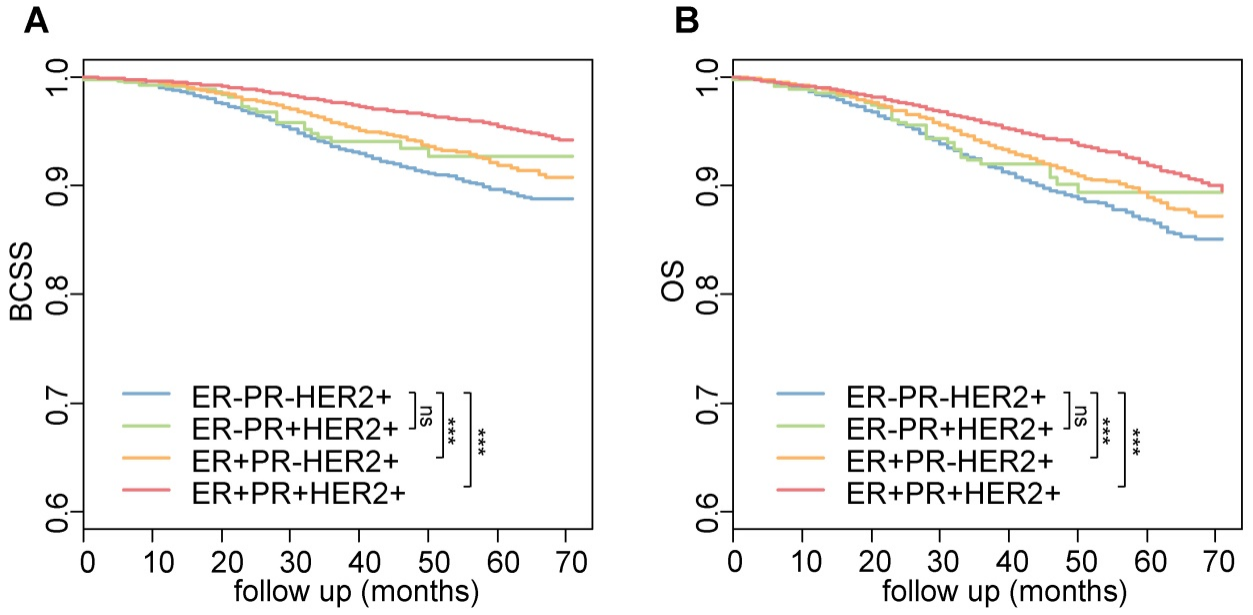
** (A) BCSS and (B) OS of HER2-positive breast cancers according to ER and PR status.** *P* values are calculated using the log-rank test. ***, *P* < 0.001; **, *P* < 0.01; *, *P* < 0.05; ns, not significant. Abbreviations: BCSS: breast cancer-specific survival; OS: overall survival.

We next compared the survival difference among the four groups. Compared with patients with ER-PR-HER2+ breast cancer, those with TPBC or ER+PR-HER2+ breast cancer had significantly better BCSS and OS (all log-rank *P* < 0.001) (Figure 1). After adjusting for patient age, race, T category, N category, tumor grade and surgery type in multivariate analyses, TPBC (BCSS, HR = 0.49, 95% CI: 0.43-0.57, *P* < 0.001; OS, HR = 0.67, 95% CI: 0.60-0.76, *P* < 0.001) and ER+PR-HER2+ breast cancer (BCSS, HR = 0.79, 95% CI: 0.67-0.93, *P* = 0.004; OS, HR = 0.82, 95% CI: 0.72-0.94, *P* = 0.005) were still associated with better BCSS and OS than ER-PR-HER2 breast cancer (Table 1). The survival difference between ER-PR+HER2+ breast cancer and ER-PR-HER2+ breast cancer was not statistically significant in either univariate or multivariate analysis.

In summary, according to ER and PR status, HER2-positive breast cancers can be divided into four groups with different clinicopathologic features. TPBCs and ER+PR-HER2+ breast cancers were associated with a better prognosis than ER-PR-HER2+ breast cancers.

### Genomic analyses revealed a lower HER2 expression level in TPBCs than in ER-PR-HER2+ breast cancers

**Figure 2:**
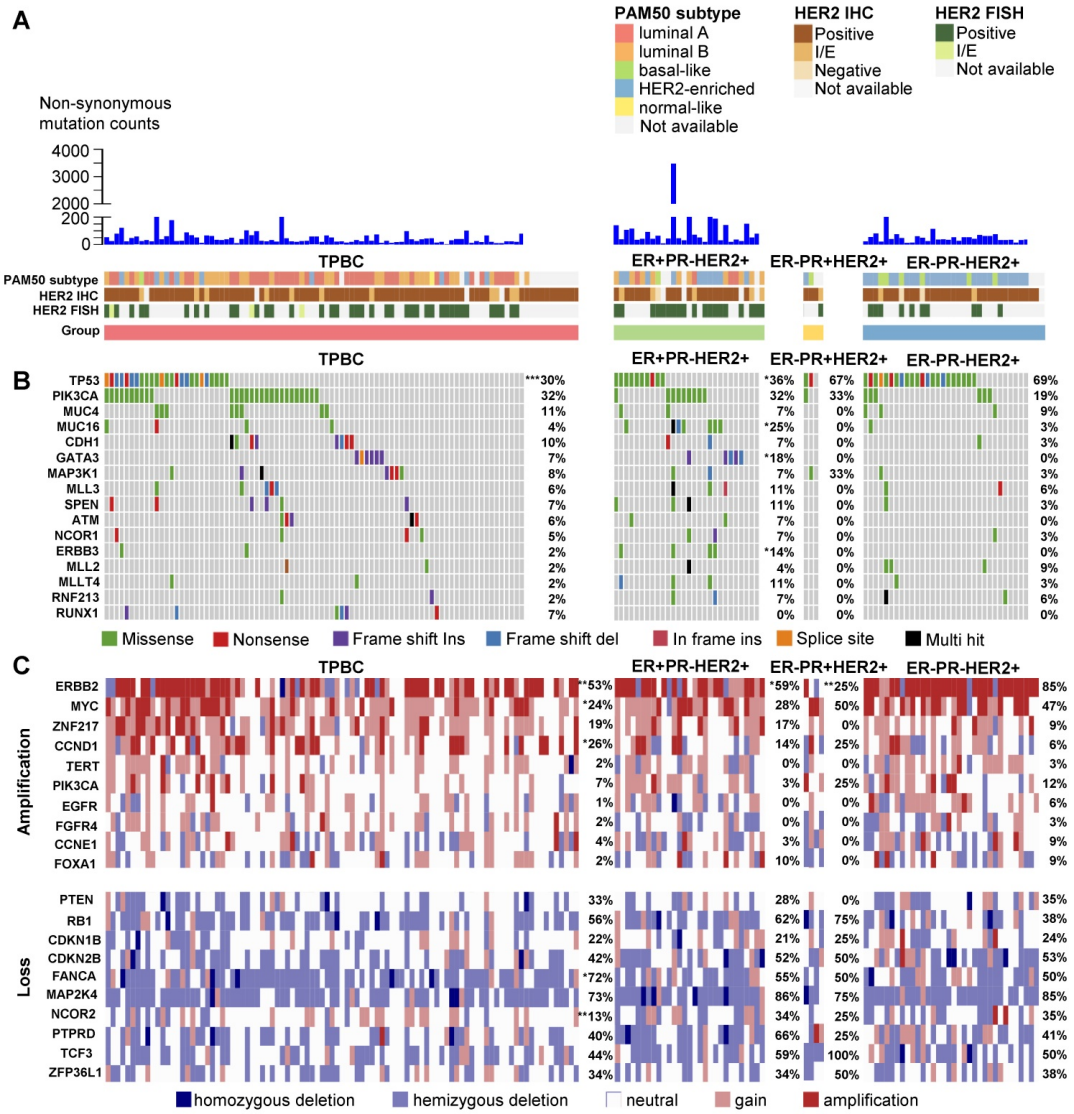
** The genomic landscape of HER2-positive breast cancers from the TCGA dataset according to ER and PR status. (A)** 162 HER2-positive samples are classified into four groups according to the IHC-based ER and PR status. Clinical and molecular features are annotated below. The mutation data are available for 147 of 162 HER2-positive cases. **(B)** Known cancer-related genes mutated in at least 4% of the patients withmutation data. The non-synonymous mutation rates of these genes in each group are listed on the right. **(C)** Known cancer-related genes located in significant GISTIC 2.0 peaks with a q value < 0.05 (The CNA events are defined according to the discrete copy number calls provided by GISTIC 2.0: -2 = homozygous deletion; -1 = hemizygous deletion; 0 = neutral; 1 = gain; 2 = amplification). Homozygous and hemizygous deletion are collectively called gene loss. The amplification or loss rates of these genes in each group are listed on the right. Differences in the rates of gene mutation, amplification and loss are compared between the ER-PR-HER+ group and each of the other three groups. *P* values are calculated using the chi-square test or Fisher's exact test. ***, *P* < 0.001; **, *P* < 0.01; *, *P* < 0.05. Abbreviations: I/E: Indeterminate or Equivocal; TPBC: triple-positive breast cancer; IHC: immunohistochemistry; FISH: fluorescence in situ hybridization.

To provide deeper insight into the biological nature of TPBCs, we investigated the genomic landscape of HER2-positive breast cancers according to ER and PR status using the TCGA data. Whole-exome sequencing data were available for 147 of the 162 HER2-positive breast cancers. Overall, 12,236 somatic mutations were identified, including 11,106 single-nucleotide variants (SNVs) and 1,130 insertions or deletions (indels) (Figure 2A). TPBCs harbored a median of 31.5 nonsynonymous somatic mutations per tumor, while ER+PR-HER2+, ER-PR+HER2+ and ER-PR-HER2+ breast cancers harbored 50.5, 40 and 40.5 nonsynonymous somatic mutations per tumor, respectively. *TP53* (40%) was the most frequently mutated gene, followed by *PIK3CA* (29%), *MUC4* (10%), *MUC16* (7%) and *CDH1* (7%). Of interest, TPBCs showed a significantly lower *TP53* mutation rate than ER-PR-HER2+ breast cancers (30% vs. 69%, *P* < 0.001). In addition, *MUC16*, *GATA3* and *ERBB3* mutations were strongly associated with the ER+PR-HER2+ phenotype (Figure 2B).

We next examined the somatic copy number alterations (CNAs) of reported cancer-related genes (Figure 2C) 13, 14. *ERBB2* was the most frequently affected gene by somatic CNAs (60%). Of note, TPBCs showed significantly lower rates of *ERBB2* and *MYC* amplification than ER-PR-HER2+ breast cancers (*ERBB2*: 53% vs. 85%, *P* = 0.001; *MYC*: 24% vs. 47%, *P* = 0.013). The frequency of *CCND1* amplification and *FANCA* loss was higher in TPBCs than in ER-PR-HER2+ breast cancers (*CCND1*: 26% vs. 6%, *P* = 0.013; *FANCA*, 72% vs. 50%, *P* = 0.023). In addition, ER+PR-HER2+ breast cancers also exhibited a lower *ERBB2* amplification rate (59% vs. 85%, *P* = 0.017).

**Figure 3:**
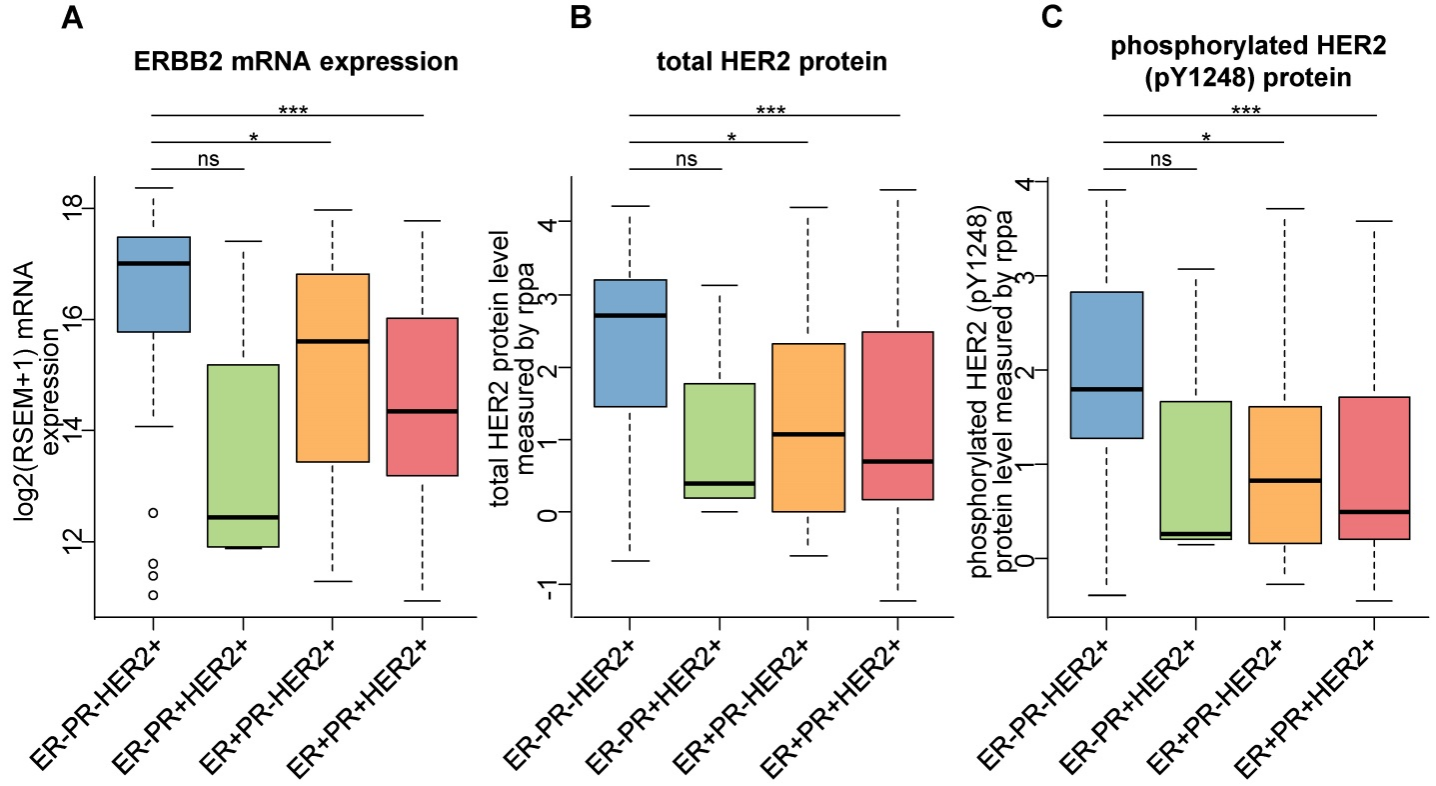
** Low levels of HER2 expression and activation in TPBC compared with ER-PR-HER2+ breast cancers. (A)** ERBB2 mRNA expression in HER2-positive breast cancers according to ER and PR status. **(B)** Total HER2 protein levels in HER2-positive breast cancers according to ER and PR status. **(C)** Phosphorylated HER2 (pY1248) protein levels in HER2-positive breast cancers according to ER and PR status. *P* values are calculated using Student's t test. ***, *P* < 0.001; **, *P* < 0.01; *, *P* < 0.05; ns, not significant. Abbreviation: TPBC: triple-positive breast cancer; rppa: reverse-phase protein array.

The different *ERBB2* amplification rates among the four groups inspired us to concentrate on the HER2 expression levels of HER2-positive breast cancers according to ER and PR status. We found that the ERBB2 mRNA expression was significantly lower in TPBCs than in ER-PR-HER2+ breast cancers (Figure 3A). Besides, both total HER2 protein and phosphorylated HER2 protein levels were significantly lower in TPBCs than in ER-PR-HER2+ breast cancers (Figure 3B-C). All these data suggested a relatively low level of HER2 expression in TPBCs compared with ER-PR-HER2+ breast cancers.

In summary, genomic analyses revealed different molecular features of TPBCs from ER-PR-HER2+ breast cancers. In particular, TPBCs showed a lower HER2 expression level than ER-PR-HER2+ breast cancers.

### Intrinsic molecular classification of TPBCs

**Figure 4:**
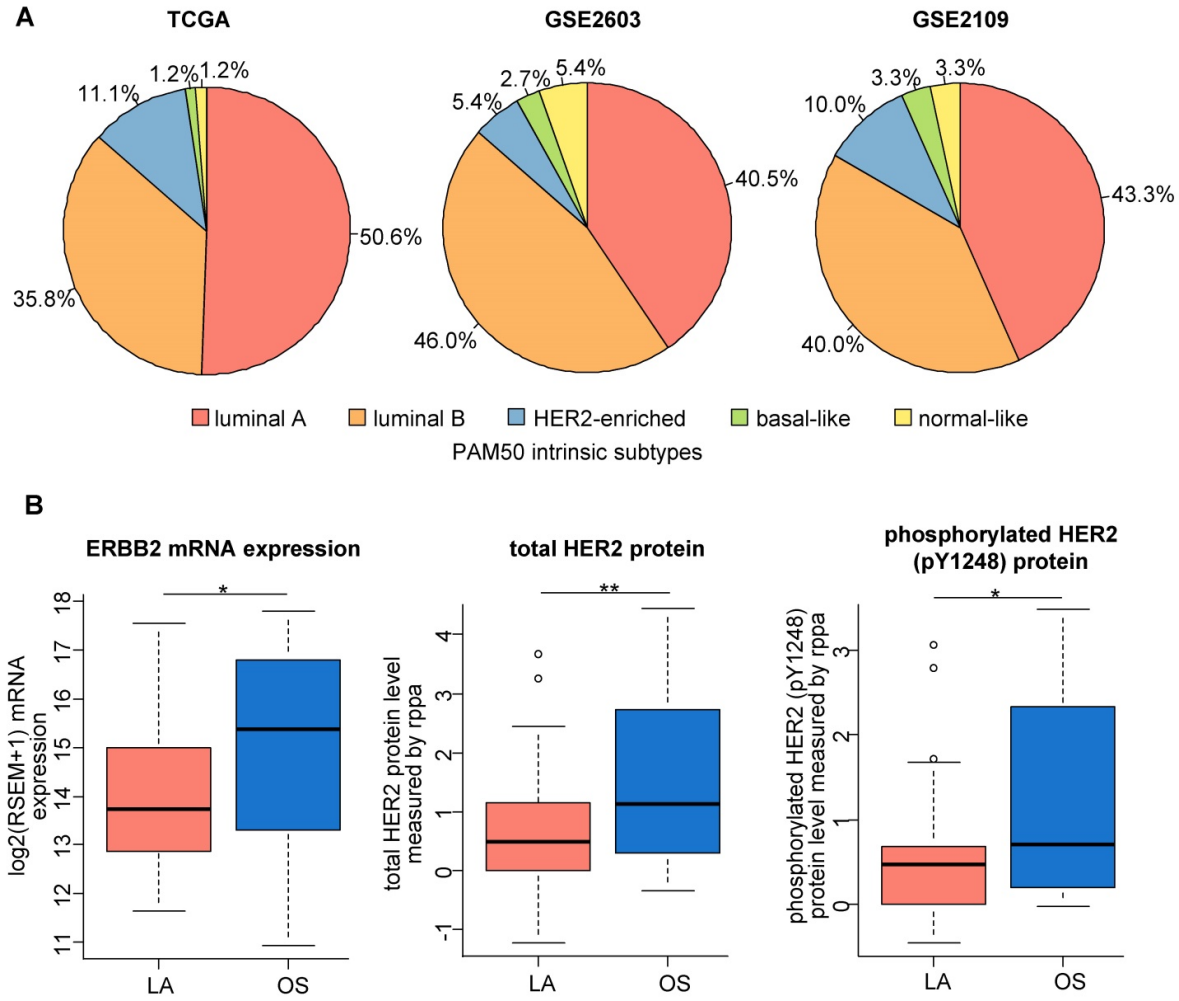
** Subtyping of TPBCs according to PAM50 intrinsic classification. (A)** PAM50 intrinsic classification of TPBCs from the TCGA, GSE2603 and GSE2109 datasets. **(B)** Low HER2 expression level of the luminal A subtype among TPBCs. *P* values are calculated using Student's t test. ***, *P* < 0.001; **, *P* < 0.01; *, *P* < 0.05. Abbreviations: TPBC: triple-positive breast cancer; LA: luminal A; OS: other subtypes; rppa: reverse-phase protein array.

Intrinsic molecular subtyping of breast cancer is essential for understanding the biological features of this disease and for making treatment choices. However, few studies have examined the intrinsic subtyping and the molecular essence of TPBCs. Thus, we explored the distribution of PAM50 intrinsic subtypes of TPBCs from the TCGA, GSE2603 and GSE2109 cohorts (Figure 4A). Surprisingly, the luminal A intrinsic subtype accounted for more than 40% of TPBCs in all three cohorts. The percentages of luminal A intrinsic subtype in TPBCs (TCGA, 50.6%; GSE2603, 40.5%; GSE2109, 43.3%) were much higher than those in ER+PR-HER2+ (TCGA, 16.0%; GSE2603, 15.4%; GSE2109, 0%) and ER-PR-HER2+ (TCGA, 0%; GSE2603, 0%; GSE2109, 9.1%) breast cancers. Based on the intrinsic molecular classification results, we further explored the HER2 expression levels in different intrinsic subtypes using the TCGA data. The ERBB2 mRNA expression, total HER2 protein level and phosphorylated HER2 protein level were all lower in the luminal A subtype than in the other subtypes (Figure 4B).

We also compared the prognosis of TPBCs of the luminal A subtype and the other subtypes using the TCGA cohort. We observed a tendency of better DFS and OS in TPBCs of the luminal A subtype than in those of the other subtypes, but the difference did not reach statistical significance due to the small sample size (DFS: log-rank *P* = 0.106, OS: log-rank *P* = 0.088; Figure S1). The differences in clinicopathologic characteristics between the luminal A subtype and the other subtypes were not significant (Table S2).

In summary, TPBCs comprised considerable luminal A intrinsic subtype breast cancers, which may be associated with a relatively good prognosis and a low HER2 expression level compared with the other intrinsic subtypes.

### Identification of luminal A-like TPBCs and its clinical implications

The PAM50 intrinsic subtyping should be regarded as an important reference to the prognostic evaluation and therapeutic decision-making in patients with TPBC. We aimed to develop a clinically feasible method to identify luminal A subtype TPBCs using immunohistochemical markers (Figure S2). We first identified the differentially expressed genes between the luminal A subtype and the other subtypes using the TCGA dataset. These genes were further filtered and validated in the GSE2603 and GSE2109 datasets (Tables S3-S4).

**Figure 5:**
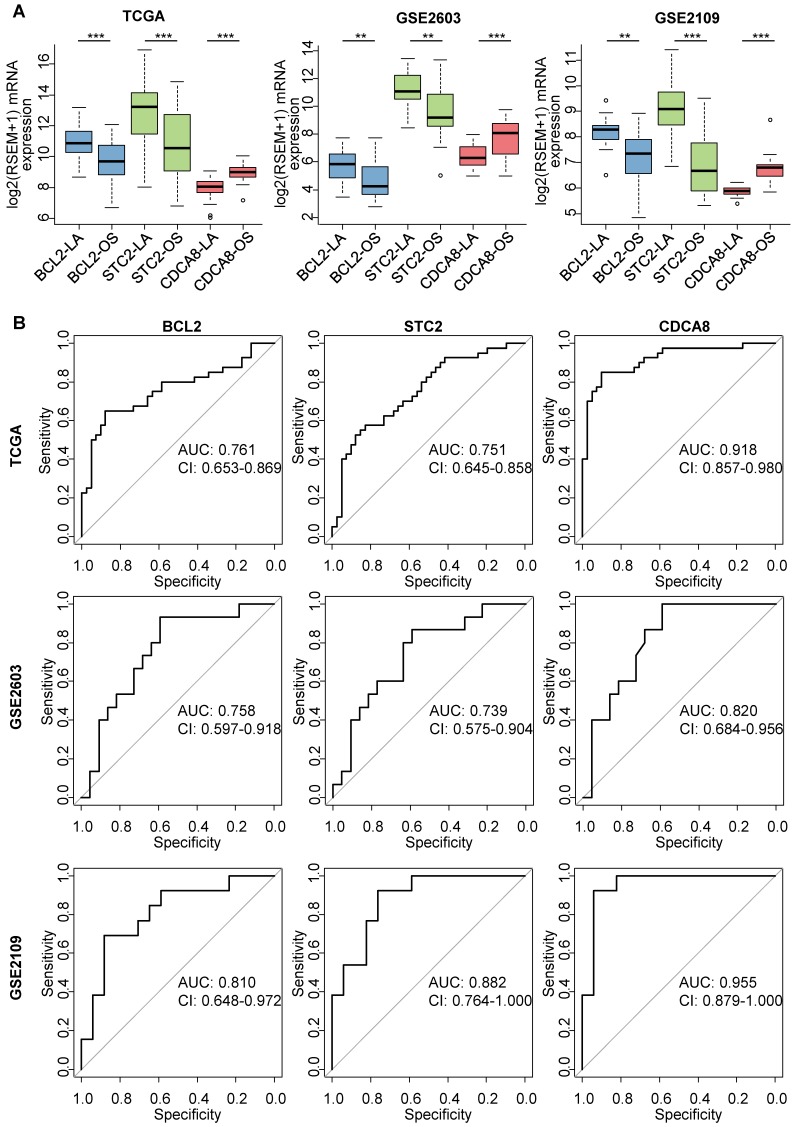
** Identification of luminal A subtype TPBCs by the mRNA expression of BCL2, STC2 and CDCA8. (A)** Expression of BCL2, STC2 and CDCA8 between TPBCs of the luminal A subtype and those of the other subtypes in the TCGA, GSE2603 and GSE2109 cohorts. *P* values are calculated using Student's t test. ***, *P* < 0.001; **, *P* < 0.01; *, *P* < 0.05. **(B)** ROC curves of using the mRNA expression of BCL2, STC2 and CDCA8 to identify luminal A subtype TPBCs. Abbreviations: TPBC: triple-positive breast cancer; LA: luminal A; OS: other subtypes; ROC: receiver operating characteristic; AUC: area under the curve; CI: confidence interval.

We next tested the relationship between the mRNA expression and protein expression of the remaining genes and retained those with a correlation coefficient of > 0.5. Finally, we conducted receiver operating characteristic (ROC) analysis to assess the accuracy of using the mRNA expression of retained genes to identify the luminal A subtype. According to the area under the curve (AUC) in the TCGA dataset, we selected STC2, BCL2 and CDCA8 (Tables S5-S6) as the immunohistochemical markers. STC2 and BCL2 were highly expressed in the luminal A subtype compared with the other subtypes, while CDCA8 was expressed at a relatively low level in the luminal A subtype (Figure 5A). A significant positive correlation was observed between each gene's protein expression and mRNA expression (Figure S3). The AUC of using BCL2 expression to identify the luminal A subtype was 0.761 in TCGA, 0.758 in GSE2603 and 0.810 in GSE2109. The AUC of using STC2 expression to identify the luminal A subtype was 0.751 in TCGA, 0.739 in GSE2603 and 0.882 in GSE2109. The AUC of using CDCA8 expression to identify the luminal A subtype was 0.918 in TCGA, 0.820 in GSE2603 and 0.955 in GSE2109 (Figure 5B). We also investigated whether the expression of ESR1, PGR and ERBB2 could be used to identify luminal A subtype TPBCs and found that the accuracy was far inferior to that of our selected genes (Figures S4-S5).

**Figure 6:**
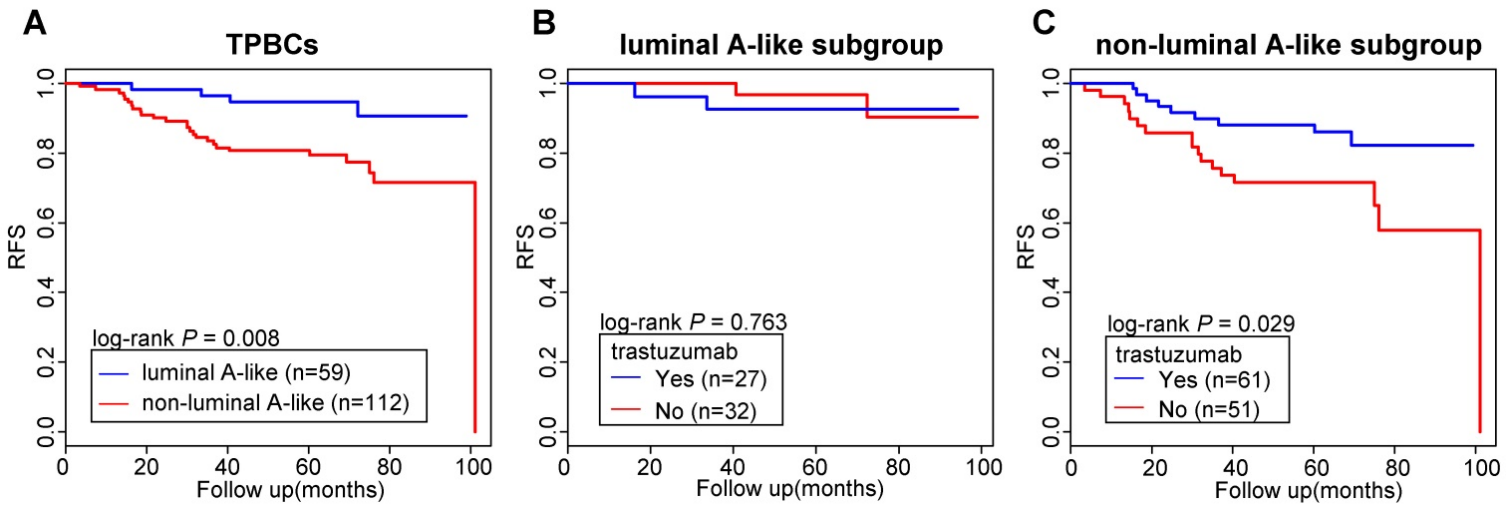
** Clinical implications of luminal A-like TPBCs. (A)** Difference in relapse-free survival between luminal A-like and non-luminal A-like TPBCs. **(B)** Comparison of relapse-free survival between patients treated with trastuzumab and those not treated with trastuzumab in the luminal A-like subgroup. **(C)** Comparison of relapse-free survival between patients treated with trastuzumab and those not treated with trastuzumab in the non-luminal A-like subgroup. *P* values are calculated using the log-rank test. Abbreviations: TPBC: triple-positive breast cancer; RFS: relapse-free survival.

Next, we validated the clinical implications of these three genes in the FUSCC cohort by performing immunohistochemical detection to identify a luminal A-like subgroup of TPBCs. According to the immunohistochemical staining results (Figure S6), we classified 171 TPBC tumors into a luminal A-like subgroup (n=59) and a non-luminal A-like subgroup (n=112) (Table S7). Tumors were classified into the luminal A-like subgroup if they were CDCA8-negative and positive for at least one of BCL2 and STC2, while those showing other immunohistochemical staining results were placed into the non-luminal A-like subgroup. Patients with luminal A-like TPBC showed significantly better RFS than those with non-luminal A-like TPBC (log-rank *P* = 0.008) (Figure 6A). In the multivariate survival analysis, the luminal A-like subgroup was still associated with better relapse-free survival (HR = 0.33, 95% CI: 0.11-0.97, *P* = 0.045) (Table S8).

Of note, in the non-luminal A-like subgroup, patients treated with trastuzumab had significantly better RFS than those not treated with trastuzumab (log-rank *P* = 0.029), while in the luminal A-like subgroup, there was no difference in RFS between patients treated with trastuzumab and those not treated with trastuzumab (log-rank *P* = 0.763) (Figure 6B-C, Figure S7).

In summary, we found that the mRNA expression of STC2, BCL2 and CDCA8 can be used to identify luminal A subtype TPBCs. Based on this result, we developed an IHC-based method incorporating these three genes to identify a luminal A-like subgroup of TPBCs and demonstrated that patients with luminal A-like TPBC had a better prognosis and benefited less from trastuzumab therapy.

## Discussion

In this study, we systemically studied the clinical and molecular features of TPBCs. We demonstrated that patients with TPBC had a significantly better prognosis than those with ER-PR-HER2+ breast cancer. We also found that TPBCs exhibited a relatively low HER2 expression level compared with ER-PR-HER2+ breast cancers. We further unraveled the intrinsic molecular classification of TPBCs and found that a considerable percentage of TPBCs were classified as the luminal A subtype that showed an even lower HER2 expression level. Finally, we developed a practical method based on the immunohistochemical detection of STC2, BCL2 and CDCA8 to identify a luminal A-like subgroup of TPBCs that was associated with a relatively good prognosis and reduced benefit from trastuzumab.

Compared with HER2-negative breast cancers, HER2-positive breast cancers are more aggressive and have been associated with a poorer prognosis before the introduction of HER2-targeted therapy. HER2-positive breast cancers can be further classified into ER+PR+HER2+, ER+PR-HER2+, ER-PR+HER2+ and ER-PR-HER2+ breast cancers according to ER and PR status. Taking advantage of the SEER database, we investigated the clinicopathologic characteristics and prognoses of these four groups. We found that compared with ER-PR-HER2+ breast cancers, TPBCs showed a lower tumor stage and grade and were independently associated with longer BCSS and OS. These results indicated that TPBCs were less aggressive than ER-PR-HER2+ breast cancers.

We next examined the molecular features of TPBCs and compared them with those of other HER2-positive subgroups. We revealed that TPBCs may have different driver events from ER-PR-HER2+ breast cancers, including a lower *TP53* mutation rate, a lower *ERBB2* amplification rate and a higher *CCND1* amplification rate. Among these events, we concentrated on *ERBB2* amplification, which has been associated with tumor sensitivity to HER2-targeted therapy 15. We further compared the differences in HER2 mRNA and protein expression between TPBCs and ER-PR-HER2+ breast cancers. All these analyses suggested a lower HER2 expression level in TPBCs.

As a clinically defined entity, TPBC is heterogeneous in terms of its intrinsic molecular subtypes. Previous studies have shown that nearly 30% of HR+/HER2+ breast cancers were classified as the luminal A subtype according to intrinsic molecular classification 12, 16. By contrast, the luminal A intrinsic subgroup accounted for more than 40% of the TPBCs in all three cohorts with intrinsic molecular classification results in our study. These luminal A subtype TPBCs showed even lower levels of HER2 mRNA and protein expression than TPBCs of the other intrinsic subtypes. This result suggested that luminal A subtype TPBCs might be driven primarily by HR signaling pathways rather than the HER2 signaling pathway 5, 17. In addition, patients with luminal A subtype TPBC tended to have a better prognosis. Thus, we inferred that luminal A subtype TPBCs represent a special subgroup of HER2-positive breast cancers with a particularly low level of HER2 expression and a favorable prognosis. This subgroup of TPBCs might benefit less from HER2-targeted therapy.

The identification of luminal A subtype TPBCs may be essential to guide individualized treatment of TPBC patients. By analyzing the gene expression profiling data, we demonstrated that the mRNA expression of STC2, BCL2 and CDCA8 can be used to identify luminal A subtype TPBCs. According to previous studies, both STC2 and BCL2 are estrogen-responsive genes that are upregulated in luminal breast cancers and correlate with a better prognosis 18-22. CDCA8 is a critical regulator of mitosis and cell division and is associated with cancer growth and progression 23. All these three markers have been detected by IHC in the previous studies 20, 24, 25. We detected these three markers by IHC in 171 TPBCs from the FUSCC cohort and identified a luminal A-like subgroup of TPBCs. Patients with luminal A-like TPBC had a better prognosis and benefited less from adjuvant trastuzumab. These results suggested that it might be possible for some patients with luminal A-like TPBC to be treated with de-escalated trastuzumab therapy. Vici et al also explored the efficacy of trastuzumab in TPBCs 26. They found that increased expression of ER was associated with reduced trastuzumab benefit and that this benefit tended to disappear in patients whose tumors expressed ER in > 50% of cells. However, our data suggested that the accuracy of using ER expression to identify the luminal A intrinsic subtype was far inferior to that of our selected markers.

Our study has some limitations. First, we did not perform intrinsic molecular subtyping of the TPBCs in the FUSCC cohort; therefore, we were unable to examine the accuracy of our IHC-based method for identifying the luminal A intrinsic subtype. Nevertheless, based on the treatment and follow-up data, we observed that patients with luminal A-like TPBC identified by our IHC-based method had a relatively better prognosis and benefited less from trastuzumab therapy. These results demonstrated the clinical implications of our IHC-based method. Second, we were unable to obtain data on HER2-targeted therapy from the SEER and TCGA datasets; therefore, we could not directly compare the efficacy of HER2-targeted therapy between TPBCs and other HER2-positive subgroups or between TPBCs of the luminal A intrinsic subtype and TPBCs of the other subtypes. Third, since there are only a very small number of patients in the FUSCC cohort who did not receive chemotherapy due to old age, we are unable to analyze whether it is possible for patients with luminal A-like TPBC to be treated with de-escalated chemotherapy.

In conclusion, compared with ER-PR-HER2+ breast cancers, TPBCs are less aggressive and show a lower HER2 expression level. A considerable proportion of TPBCs are luminal A subtype breast cancers according to the intrinsic molecular classification. Evaluating the expression of STC2, BCL2 and CDCA8 via IHC can help identify a luminal A-like subgroup of TPBCs, which has a relatively better prognosis and benefits less from trastuzumab therapy.

## Supplementary Material

Supplementary materials and methods, figures and tables.

## Figures and Tables

**Table 1:** Multivariate analyses of breast cancer-specific survival (BCSS) and overall survival (OS) using Cox proportional hazards models in the SEER cohort.

		BCSS			OS	
Variables		HR (95% CI)	*P*		HR (95% CI)	*P*
Age at diagnosis						
	≤ 50 years	Reference	-		Reference	-
	> 50 years	1.83 (1.59-2.10)	< 0.001		2.64 (2.32-3.01)	< 0.001
Race						
	White	Reference	-		Reference	-
	Black	1.29 (1.10-1.52)	0.002		1.15 (1.00-1.32)	0.052
	Others	0.56 (0.44-0.71)	< 0.001		0.58 (0.48-0.70)	< 0.001
Grade						
	1 or 2	Reference	-		Reference	-
	3 or UD	1.43 (1.24-1.65)	<0.001		1.23 (1.10-1.37)	< 0.001
T category						
	T1	Reference	-		Reference	-
	T2-4	2.67 (2.27-3.13)	< 0.001		2.01 (1.79-2.26)	< 0.001
N category						
	N0	Reference	-		Reference	-
	N1-3	2.63 (2.28-3.04)	< 0.001		1.75 (1.57-1.95)	< 0.001
HER2+ subgroup						
	ER-PR-HER2+	Reference	-		Reference	-
	ER-PR+HER2+	0.77 (0.51-1.17)	0.222		0.88 (0.63-1.24)	0.477
	ER+PR-HER2+	0.79 (0.67-0.93)	0.004		0.82 (0.72-0.94)	0.005
	ER+PR+HER2+	0.49 (0.43-0.57)	< 0.001		0.67 (0.60-0.76)	< 0.001
Surgery						
	Lumpectomy	Reference	-		Reference	-
	Mastectomy	1.54 (1.34-1.78)	< 0.001		1.42 (1.27-1.58)	< 0.001

Abbreviations: BCSS: breast cancer-specific survival; OS: overall survival; HR: hazard ratio; CI: confidence interval; UD: undifferentiated.

## Abbreviations

AUC: area under the curve
BCSS: breast-cancer-specific survival
CC: correlation coefficient
CNA: copy number alteration
ER: estrogen receptor
FUSCC: Fudan University Shanghai Cancer Center
HER2: human epidermal growth factor receptor 2
IHC: immunohistochemistry
OS: overall survival
PR: progesterone receptor
RFS: relapse-free survival
ROC: receiver operating characteristic
SEER: Surveillance, Epidemiology, and End Results
TCGA: The Cancer Genome Atlas
TPBC: triple-positive breast cancer
