# Supplementary material for: Molecular portraits and trastuzumab responsiveness of estrogen receptor-positive, progesterone receptor-positive, and HER2-positive breast cancer
Source: Theranostics. 2019 Jul 9;9(17):4935–45. doi: 10.7150/thno.35730 (PMC6691389; doi:10.7150/thno.35730)
Supplement: Supplementary file 1 — Supplementary materials and methods, figures and tables. [file thnov09p4935s1.pdf]

Supplementary Materials:

Supplementary Methods

Supplementary Tables: Tables S1-S9

Supplementary Figures: Figures S1-S7

Supplementary Methods

1. Study cohorts

Our study comprised five cohorts. The first cohort consisted of 32,056 patients with human epidermal growth factor receptor 2 (HER2)-positive breast cancer identified from the Surveillance, Epidemiology and End Results (SEER) database. The inclusion criteria were as follows: female, aged between 18 and 90 years, year of diagnosis from 2010 to 2015, breast cancer as the first and only malignant cancer diagnosis, unilateral breast cancer, HER2-positive breast cancer, American Joint Committee on Cancer (AJCC) stages I-III, and having received a mastectomy or a lumpectomy as primary surgical treatment. We excluded patients who lacked a histologically confirmed diagnosis and those identified by death certificate or autopsy. We used this cohort to examine the clinicopathologic features and prognoses of HER2-positive breast cancers according to the estrogen receptor (ER) and progesterone receptor (PR) status.

The second cohort included 162 HER2-positive breast cancer patients from The Cancer Genome Atlas (TCGA). The clinical data of these patients were extracted from the “data_bcr_clinical_data_patient” file downloaded from cBioPortal (http://www.cbioportal.org). The inclusion criteria were as follows: female, HER2-positive breast cancer and AJCC stages I-III. The HER2 status

was determined according to the newest ASCO/CAP guidelines [1]. An intrinsic molecular subtype was assigned to the tumor of each patient by the PAM50 classifier [2, 3]. We performed three parts of analysis based on the data from this cohort. First, we investigated the genomic landscape and the HER2 expression level of HER2-positive breast cancers according to the ER and PR status. Second, we analyzed the intrinsic molecular classification of ER+PR+HER2+ breast cancers (triple-positive breast cancers, TPBCs) and examined the HER2 expression level of TPBCs according to the intrinsic molecular classification. Third, we identified differentially expressed genes between the luminal A subtype and the other subtypes, and assessed the accuracy of using certain genes to identify the luminal A subtype.

The third and fourth cohorts were from two publicly available microarray datasets (GSE2603 and GSE2109), which included 37 and 30 patients with TPBC, respectively. The gene expression data were normalized by Haibe-Kains et al [4]. Hybridization probes were mapped to the Entrez Gene ID as described by Shi et al [5]. When multiple probes mapped to the same gene ID, the one with the highest variance was used. Tumors of these patients were classified into PAM50 intrinsic subtypes according to the description by Haibe-Kains et al [2, 4]. We used these two cohorts to further filter and validate the differentially expressed genes identified in the TCGA cohort.

The fifth cohort was a prospective observational study cohort. A total of 171 consecutive TPBC patients treated at Fudan University Shanghai Cancer Center (FUSCC) between 2007 and 2014 were enrolled according to the following criteria: female, aged between 18 and 90 years, breast cancer as the

first and only malignant cancer diagnosis, unilateral breast cancer, histologically confirmed invasive carcinoma of the ER+PR+HER2+ phenotype, without metastatic loci at diagnosis, and having available formalin-fixed, paraffin-embedded (FFPE) surgical specimens. The exclusion criteria were as follows: breast carcinoma in situ, and having received any type of treatment before surgery. ER, PR and HER2 status were independently confirmed by two experienced pathologists based on immunohistochemical analysis and in situ hybridization. We used a cutoff of ≥ 1% positive tumor cells to define ER positivity and PR positivity and determined HER2 status according to the newest ASCO/CAP guidelines. Follow-up was completed in June 2018. The median length of follow-up was 66.2 months (interquartile range, 52.8 to 75.9 months). In this cohort, based on the immunohistochemical detection of STC2, BCL2 and CDCA8, we identified a luminal A-like subgroup of TPBCs and analyzed its prognosis and trastuzumab responsiveness. Our study was approved by the independent Ethics Committee/Institutional Review Board of FUSCC. Each patient provided written informed consent.

2. Bioinformatics analysis

2.1 Somatic mutation analysis

Somatic mutation data of the TCGA cohort were extracted from the "data_mutations_extended" file downloaded from cBioPortal (http://www.cbioportal.org). We identified the known cancer-related genes [6, 7] mutated at a frequency of ≥ 4% in HER2+ breast cancers. The difference in mutation rates between the ER-PR-HER2+ group and each of the other three groups (ER-PR+HER2+, ER+PR-HER2+ and ER+PR+HER2+) was compared

using Chi-square test or Fisher's exact test.

2.2 Somatic copy number analysis

Segmented copy number alteration (CNA) data of the TCGA cohort were extracted from the file "gdac.broadinstitute.org_BRCATP.CopyNumber_Gistic2.Level_4.2016012800.0.0" downloaded from Broad GDAC Firehose (http://gdac.broadinstitute.org). Putative copy number calls were determined using Genomic Identification of Significant Targets in Cancer (GISTIC 2.0) [7]. The CNA events were defined according to the discrete copy number calls provided by GISTIC 2.0: -2 = homozygous deletion; -1 = hemizygous deletion; 0 = neutral; 1 = gain; 2 = amplification. The difference in CNA event rates was compared between ER-PR-HER2+ breast cancers and each of the other three HER2-positive breast cancer subgroups (ER-PR+HER2+, ER+PR-HER2+ and ER+PR+HER2+) using Chi-square test or Fisher's exact test.

2.3 RNA-Seq analysis

The gene-level RSEM (RNA-Seq by Expectation-Maximization) data of the TCGA cohort were extracted from the "data_RNA_Seq_v2_expression_median" file downloaded from cBioPortal (http://www.cbioportal.org). The RSEM values were log2-transformed after adding a constant of 1 to all values. Raw count data were obtained from the GDC portal (https://portal.gdc.cancer.gov), which is used for differential expression analysis.

2.4 Protein expression analysis

Two kinds of protein expression data of the TCGA cohort are used in our study. The first is log2-transformed protein expression data measured by reverse-phase protein array (rppa), which are extracted from the "data_rppa" file downloaded from cBioPortal (http://www.cbioportal.org). These data cover 892 TCGA cases but only 183 genes. The second is protein expression data measured with mass spectrometry by the Clinical Proteomic Tumor Analysis Consortium (CPTAC), which are extracted from the "data_protein_quantification" file downloaded from cBioPortal (http://www.cbioportal.org). These data cover 11266 genes but only 74 TCGA cases. The rppa data were used to compare the levels of total HER2 protein and phosphorylated HER2 (pY1248) protein of different HER2-positive subgroups (ER-PR-HER2+, ER-PR+HER2+, ER+PR-HER2+ and ER+PR+HER2+). The mass spectrometry data were not used in this analysis because they are available for only 14 HER2-positive cases. The mass spectrometry data of all TCGA cases were used to assess the correlation between the genes' protein expression and mRNA expression when we selected the luminal A-related genes. The rppa data were not used in this analysis because they covered very few genes and a lot of candidate genes were not covered.

3. Selection of genes to identify luminal A subtype TPBCs (Figure S2)

First, we focused on 81 patients with TPBC in the TCGA cohort and identified differentially expressed genes between the luminal A intrinsic subtype

and the other subtypes using the R package “limma” with its *voom* method [8] (|fold change| ≥ 2, adjusted P value < 0.05). Then, with data obtained from GSE2603 and GSE2109, we performed Student’s t test to compare the expression of these genes between the luminal A subtype and the other subtypes and retained those with FDR < 0.05 (Tables S4-S5). Next, we assessed the correlation of the mRNA expression with the protein expression of the remaining genes using TCGA dataset and excluded those with a correlation coefficient ≤ 0.5. Finally, we conducted receiver operating characteristic (ROC) analysis in the TCGA, GSE2603 and GSE2109 cohorts to test the accuracy of using the candidate genes to identify the luminal A subtype and ordered these genes by the area under the curve (AUC) in the TCGA cohort (Tables S6-S7). Two highly expressed genes in the luminal A subtype and one lowly expressed gene in the luminal A subtype with the highest AUC in the TCGA cohort were selected.

4. Immunohistochemical staining and results interpretation

For patients in the FUSCC cohort, we performed immunohistochemical staining on tissue microarrays (TMAs) to evaluate the expression of STC2, BCL2 and CDCA8. The FFPE tumor specimens were obtained for each patient and were used to construct TMAs. For the specimen of each patient, two or three representative areas were selected from hematoxylin and eosin-stained slides, and the corresponding cores (1 mm in diameter) were extracted from FFPE blocks for the construction of TMAs. The TMA slides were deparaffinized

with dimethylbenzene and rehydrated through a series of graded alcohols. Antigen retrieval was performed by heating slides for 15 min in Tris-EDTA buffer (pH=9.0) at 95°C. The slides were incubated with the primary antibody at 4°C overnight and then incubated with the secondary antibody for 30 min at 37°C. 3,3′-Diaminobenzidine was used to detect and visualize the staining. The primary antibodies used were Stanniocalcin 2 antibody (ProteinTech, 10314-1-AP, 1:400 dilution), Bcl-2 antibody (Abcam, ab32124, 1:200 dilution), and CDCA8 antibody (ProteinTech, 12465-1-AP, 1:400 dilution).

The immunohistochemical staining of all these three markers was mainly found in the cytoplasm of tumor cells. For each of the markers, almost all positively stained specimens showed diffuse staining in all the tumor cells and few specimens exhibited focal staining. Thus, we used staining intensity to measure the protein expression of these three markers [9]. The staining intensity was scored as follows: 0, negative; 1, weak; 2, moderate; and 3, strong. For each case, the corresponding TMA cores were assessed individually and an overall intensity score was calculated by averaging the intensity scores of all the corresponding cores. For each of the three markers, an overall intensity score of ≥ 2 was used to classify the tumor as positive, and an overall intensity score of < 2 was used to classify the tumor as negative. All stained TMAs were independently evaluated by two experienced pathologists who were blinded to the patients' clinical information. Discrepancies in scoring results between the two pathologists were resolved by discussion and consensus.

References

1. Wolff AC, Hammond MEH, Allison KH, Harvey BE, Mangu PB, Bartlett JMS, et al. Human Epidermal Growth Factor Receptor 2 Testing in Breast Cancer: American Society of Clinical Oncology/College of American Pathologists Clinical Practice Guideline Focused Update. J Clin Oncol. 2018; 36: 2105-22.
2. Parker JS, Mullins M, Cheang MC, Leung S, Voduc D, Vickery T, et al. Supervised risk predictor of breast cancer based on intrinsic subtypes. J Clin Oncol. 2009; 27: 1160-7.
3. Ciriello G, Gatza ML, Beck AH, Wilkerson MD, Rhie SK, Pastore A, et al. Comprehensive Molecular Portraits of Invasive Lobular Breast Cancer. Cell. 2015; 163: 506-19.
4. Haibe-Kains B, Desmedt C, Loi S, Culhane AC, Bontempi G, Quackenbush J, et al. A three-gene model to robustly identify breast cancer molecular subtypes. J Natl Cancer Inst. 2012; 104: 311-25.
5. Consortium M, Shi L, Reid LH, Jones WD, Shippy R, Warrington JA, et al. The MicroArray Quality Control (MAQC) project shows inter- and intraplatform reproducibility of gene expression measurements. Nat Biotechnol. 2006; 24: 1151-61.
6. An O, Dall'Olio GM, Mourikis TP, Ciccarelli FD. NCG 5.0: updates of a manually curated repository of cancer genes and associated properties from cancer mutational screenings. Nucleic acids research. 2016; 44: D992-9.
7. Futreal PA, Coin L, Marshall M, Down T, Hubbard T, Wooster R, et al. A census of human cancer genes. Nat Rev Cancer. 2004; 4: 177-83.
8. Law CW, Alhamdoosh M, Su S, Dong X, Tian L, Smyth GK, et al. RNA-seq analysis is easy as 1-2-3 with limma, Glimma and edgeR. F1000Research. 2016; 5.
9. Meyer HA, Tolle A, Jung M, Fritzsche FR, Haendler B, Kristiansen I, et al. Identification of stanniocalcin 2 as prognostic marker in renal cell carcinoma. Eur Urol. 2009; 55: 669-78.

Table S1. Clinicopathologic features of HER2-positive breast cancers according to ER and PR status in the SEER cohort.[a]

Characteristics	HER2+ breast cancer subgroups											
	Total		ER-PR-HER2+		ER-PR+HER2+		*P*	ER+PR-HER2+		*P*	ER+PR+HER2+		*P*
	n=32056		n=9350		n=617			n=5790			n=16299		
Age at diagnosis							0.013			< 0.001			< 0.001
≤ 50 years	10645	33.2	2893	30.9	221	35.8		1463	25.3		6068	37.2	
> 50 years	21411	66.8	6457	69.1	396	64.2		4327	74.7		10231	62.8	
Race							0.852			< 0.001			< 0.001
White	24130	75.3	6744	72.1	441	71.5		4368	75.4		12577	77.2	
Black	3902	12.2	1282	13.7	83	13.5		704	12.2		1833	11.2	
Others[b]	3806	11.9	1261	13.5	88	14.3		674	11.6		1783	10.9	
Unknown	218	0.7	63	0.7	5	0.8		44	0.8		106	0.7	
Histologic type							0.510			< 0.001			< 0.001
NST	27898	87.0	8405	89.9	549	89.0		5007	86.5		13937	85.5	
special subtype	4158	13.0	945	10.1	68	11.0		783	13.5		2362	14.5	
Grade							0.948			< 0.001			< 0.001
1 or 2	13170	41.1	2292	24.5	150	24.3		2395	41.4		8333	51.1	
3 or UD	18886	58.9	7058	75.5	467	75.7		3395	58.6		7966	48.9	
T category							0.399			< 0.001			< 0.001
T1	16205	50.6	4354	46.6	276	44.7		2976	51.4		8599	52.8	
T2-4	15851	49.4	4996	53.4	341	55.3		2814	48.6		7700	47.2	
N category							0.915			< 0.001			< 0.001
N0	19376	60.4	5393	57.7	354	57.4		3582	61.9		10047	61.6	
N1-3	12680	39.6	3957	42.3	263	42.6		2208	38.1		6252	38.4	
Surgery type							0.299			< 0.001			< 0.001
Lumpectomy	15318	47.8	3990	42.7	277	44.9		2714	46.9		8337	51.2	
Mastectomy	16738	52.2	5360	57.3	340	55.1		3076	53.1		7962	48.8	
Chemotherapy							0.776			< 0.001			< 0.001
Yes	24294	75.8	7357	78.7	489	79.3		4323	74.7		12125	74.4	
No/Unknown	7762	24.2	1993	21.3	128	20.7		1467	25.3		4174	25.6	
Radiotherapy							0.157			0.753			< 0.001
Yes	15816	49.3	4415	47.2	310	50.2		2750	47.5		8341	51.2	
No/Unknown	16240	50.7	4935	52.8	307	49.8		3040	52.5		7958	48.8	

a. Data are presented as number (percentage) of patients. Differences between the ER-PR-HER2+ group and each of the other three groups are compared using Pearson's chi-square test or Fisher's exact test.

b. Including American Indian, Alaskan Native, Asian and Pacific Islander.

Abbreviations: TPBC: triple-positive breast cancer; NST: no special type; UD: undifferentiated.

Table S2. PAM50 intrinsic classification of HER2-positive breast cancers according to ER and PR status from the TCGA, GSE2603 and GSE2109 datasets.

	ER-PR-HER2+		ER-PR+HER2+		ER+PR-HER2+		TPBC	
	N	(%)	N	(%)	N	(%)	N	(%)
TCGA								
luminal A	0	0.0	0	0.0	4	16.0	41	50.6
luminal B	0	0.0	0	0.0	10	40.0	29	35.8
HER2-enriched	26	81.3	1	50.0	10	40.0	9	11.1
basal-like	6	18.8	1	50.0	1	4.0	1	1.2
normal-like	0	0.0	0	0.0	0	0.0	1	1.2
GSE2603								
luminal A	0	0.0	0	0.0	2	16.7	15	40.5
luminal B	1	2.9	0	0.0	6	50.0	17	46.0
HER2-enriched	10	28.6	0	0.0	3	25.0	2	5.4
basal-like	21	60.0	1	100.0	1	8.3	1	2.7
normal-like	3	8.6	0	0.0	0	0.0	2	5.4
GSE2109								
luminal A	2	9.1	0	-	0	0.0	13	43.3
luminal B	1	4.5	0	-	5	55.6	12	40.0
HER2-enriched	10	45.5	0	-	1	11.1	3	10.0
basal-like	9	40.9	0	-	2	22.2	1	3.3
normal-like	0	0.0	0	-	1	11.1	1	3.3

Data are presented as number (percentage) of patients.

Abbreviation: TPBC: triple-positive breast cancer.

Table S3. Clinicopathologic features of TPBCs from the TCGA, GSE2603 and GSE2109 datasets.

	TCGA (n=81)					GSE2603 (n=37)					GSE2109 (n=30)				
	luminal A (n=41)		other subtypes (n=40)			luminal A (n=15)		other subtypes (n=22)			luminal A (n=13)		other subtypes (n=17)		
	N	(%)	N	(%)	*P*	N	(%)	N	(%)	*P*	N	(%)	N	(%)	*P*
Age					0.524					0.179					-
< 50	15	(36.6)	11	(27.5)		4	(26.7)	12	(54.5)		-	-	-	-	
≥ 50	26	(63.4)	29	(72.5)		11	(73.3)	10	(45.5)		-	-	-	-	
T category					0.275					1.000					0.104
T1	10	(24.4)	5	(12.5)		2	(13.3）	4	(18.2)		6	(46.2)	5	(29.4)	
T2-4	31	(75.6)	35	(87.5)		13	(86.7)	18	(81.8)		7	(53.8)	12	(70.6)	
N category					1.000					1.000					0.705
N0	16	(39.0)	15	(37.5)		3	(20.0)	5	(22.7)		5	(38.5)	5	(29.4)	
N1-3	25	(61.0)	24	(60.0)		12	(80.0)	17	(77.3)		8	(61.5)	12	(70.6)	
Unknown	0	(0.0)	1	(2.5)		0	(0.0)	0	(0.0)		0	(0.0)	0	(0.0)	
Grade					-					-					0.433
I-II	-	-	-	-		-	-	-	-		1	(7.7)	0	(0.0)	
> II	-	-	-	-		-	-	-	-		12	(92.3)	17	(100.0)	

Data are presented as number (percentage) of patients. Differences between the luminal A subtype and the other subtypes are compared using Pearson’s chi-square test or Fisher’s exact test.

Abbreviation: TPBC: triple-positive breast cancer.

Table S4. Highly expressed genes in luminal A TPBCs compared with non-luminal A TPBCs identified by the limma package.

Genes	TCGA_logFC	TCGA_adjusted_P.value	t.test_P.value_GSE2603	t.test_FDR_GSE2603	t.test_P.value_GSE2109	t.test_FDR_GSE2109
STC2	1.94	1.92E-03	5.27E-03	3.64E-02	7.20E-05	3.10E-04
RAI2	1.50	3.82E-05	1.04E-02	4.95E-02	3.86E-04	1.34E-03
ELN	1.28	2.25E-03	7.50E-03	4.15E-02	1.14E-02	2.42E-02
MFAP4	1.23	1.86E-03	1.18E-03	1.53E-02	1.07E-02	2.29E-02
BCL2	1.18	1.19E-03	7.68E-03	4.15E-02	3.14E-03	8.19E-03
CX3CR1	1.06	3.78E-03	9.99E-03	4.87E-02	2.99E-03	7.94E-03

TCGA_logFC and TCGA_adjusted_P.value are calculated using the limma package. False discovery rates are calculated using the R function “p.adjust” for multiple testing adjustment.

Abbreviations: TPBC: triple-positive breast cancer; logFC: $\log_2$ (fold change); FDR: false discovery rate.

Table S5. Lowly expressed genes in luminal A TPBCs compared with non-luminal A TPBCs identified by the limma package.

Genes	TCGA_logFC	TCGA_adjusted_P.value	t.test_P.value_GSE2603	t.test_FDR_GSE2603	t.test_P.value_GSE2109	t.test_FDR_GSE2109
MYBL2	-1.72	1.45E-09	5.49E-03	3.65E-02	1.18E-05	7.44E-05
E2F8	-1.68	8.08E-07	7.64E-03	4.15E-02	2.88E-04	1.05E-03
UBE2C	-1.52	9.35E-09	1.78E-04	1.18E-02	5.10E-04	1.67E-03
SPAG5	-1.42	8.51E-07	7.67E-04	1.40E-02	4.24E-05	1.89E-04
TTK	-1.37	4.21E-07	1.22E-03	1.53E-02	3.02E-06	3.72E-05
SLC7A5	-1.35	1.04E-03	9.31E-03	4.65E-02	2.78E-03	7.45E-03
TPX2	-1.33	9.35E-09	3.78E-03	2.80E-02	3.11E-07	8.43E-06
NCAPG	-1.33	6.98E-08	1.07E-02	4.96E-02	1.34E-08	1.87E-06
CCNE2	-1.30	6.60E-05	3.01E-03	2.48E-02	1.12E-03	3.28E-03
CCNB2	-1.28	1.36E-07	2.70E-04	1.18E-02	1.13E-06	2.10E-05
CCNA2	-1.26	6.98E-08	1.21E-03	1.53E-02	1.15E-06	2.10E-05
HJURP	-1.26	4.91E-08	6.34E-03	3.84E-02	9.66E-06	6.38E-05
CDKN3	-1.26	5.87E-08	8.88E-04	1.48E-02	2.38E-07	7.50E-06
SKA1	-1.25	4.67E-07	7.42E-03	4.15E-02	8.88E-06	6.17E-05
MELK	-1.24	7.03E-07	1.82E-03	2.03E-02	1.13E-05	7.28E-05
CDC20	-1.18	4.15E-06	5.93E-04	1.40E-02	1.76E-06	2.80E-05
POLQ	-1.18	5.11E-06	8.61E-03	4.42E-02	1.72E-04	6.57E-04
NDC80	-1.16	2.24E-07	1.51E-03	1.78E-02	9.62E-08	5.21E-06
BUB1B	-1.15	2.70E-06	2.02E-03	2.13E-02	4.89E-06	4.91E-05
GINS1	-1.15	7.03E-06	2.18E-03	2.18E-02	2.02E-04	7.50E-04
KIF14	-1.15	8.34E-04	7.12E-04	1.40E-02	5.12E-06	4.95E-05
HMMR	-1.14	7.22E-07	6.12E-03	3.82E-02	7.15E-06	5.54E-05
RACGAP1	-1.14	3.64E-08	8.61E-03	4.42E-02	2.51E-05	1.24E-04
CDCA8	-1.12	4.35E-09	5.14E-04	1.40E-02	1.27E-05	7.50E-05
KIF2C	-1.12	1.52E-07	4.71E-04	1.40E-02	1.38E-08	1.87E-06
BIRC5	-1.11	2.19E-05	1.18E-03	1.53E-02	3.40E-05	1.59E-04
KIF18B	-1.11	5.42E-05	1.19E-04	1.18E-02	4.60E-06	4.80E-05
CCNE1	-1.10	1.20E-05	5.65E-03	3.65E-02	1.88E-05	1.00E-04
CCNB1	-1.08	2.70E-06	6.87E-05	1.18E-02	2.96E-04	1.07E-03
PRC1	-1.03	8.51E-07	2.51E-03	2.39E-02	1.39E-04	5.45E-04
PTTG1	-1.02	2.15E-05	2.94E-04	1.18E-02	6.44E-08	4.36E-06
TRIP13	-1.02	5.16E-04	6.65E-04	1.40E-02	9.33E-06	6.32E-05
MAD2L1	-1.01	5.60E-06	2.74E-03	2.39E-02	1.01E-04	4.14E-04

TCGA_logFC and TCGA_adjusted_P.value are calculated using the limma package. False discovery rates are calculated using the R function “p.adjust” for multiple testing adjustment.

Abbreviations: TPBC: triple-positive breast cancer; logFC: $\log_2$ (fold change); FDR: false discovery rate.

Table S6. Highly expressed candidate genes to identify luminal A TPBCs.

Genes	AUC_TCGA	AUC_GSE2603	AUC_GSE2109	Correlation between protein and mRNA expression correlation coefficient	Correlation between protein and mRNA expression. P.Value_correlation test
BCL2	0.761	0.758	0.810	0.669	1.33E-10
STC2	0.751	0.739	0.882	0.826	1.32E-19

Correlation coefficients and *P* values are calculated using the Pearson correlation test.

Abbreviations: TPBC: triple-positive breast cancer; AUC: area under the curve.

Table S7. Lowly expressed candidate genes to identify luminal A TPBCs.

Genes	AUC_TCGA	AUC_GSE2603	AUC_GSE2109	Correlation between protein and mRNA expression correlation coefficient	Correlation between protein and mRNA expression. P.Value_correlation test
CDCA8	0.918	0.820	0.955	0.540	1.40E-06
UBE2C	0.877	0.836	0.860	0.653	2.98E-10
KIF2C	0.876	0.788	0.982	0.762	3.36E-15
NDC80	0.871	0.821	0.959	0.764	2.47E-15
NCAPG	0.863	0.785	0.982	0.647	4.68E-10
CDC20	0.845	0.818	0.946	0.501	7.24E-06
SKA1	0.844	0.785	0.932	0.574	2.03E-05
PRC1	0.844	0.791	0.882	0.751	1.33E-14
SPAG5	0.842	0.779	0.932	0.630	4.07E-09
CCNB1	0.829	0.855	0.851	0.654	6.11E-10
GINS1	0.820	0.809	0.887	0.593	1.02E-07
MAD2L1	0.818	0.773	0.887	0.572	1.00E-07
TRIP13	0.812	0.815	0.928	0.680	2.66E-11
KIF18B	0.783	0.852	0.932	0.752	2.68E-09
SLC7A5	0.768	0.752	0.805	0.723	1.09E-12
BIRC5	0.766	0.842	0.910	0.598	3.51E-07

Correlation coefficients and *P* values are calculated using the Pearson correlation test.

Abbreviations: TPBC: triple-positive breast cancer; AUC: area under the curve.

Table S8. Clinicopathologic features of TPBCs from the FUSCC cohort.

Characteristics	FUSCC cohort				
	lumianl A-like		non-lumianl A-like		*P*
	N	(%)	N	(%)	
Age					0.411
≤ 50	28	47.5	62	55.4	
> 50	31	52.5	50	44.6	
T category					0.218
T1	27	45.8	39	34.8	
T2-4	32	54.2	73	65.2	
N category					0.071
N0	33	55.9	45	40.2	
N1-3	26	44.1	67	59.8	
Grade					0.030
I-II	42	71.2	59	52.7	
> II	17	28.8	53	47.3	
Surgery type					0.776
Lumpectomy	4	6.8	5	4.5	
Mastectomy	55	93.2	107	95.5	
Radiotherapy					0.123
Yes	14	23.7	41	36.6	
No	45	76.3	71	63.4	
Trastuzumab					0.357
Yes	27	45.8	61	54.5	
No	32	54.2	51	45.5	

Data are presented as number (percentage) of patients. Differences between the luminal A-like subgroup and the non-luminal A-like subgroup are compared using Pearson’s chi-square test or Fisher’s exact test.

Abbreviations: TPBC: triple-positive breast cancer; FUSCC: Fudan University Shanghai Cancer Center.

Table S9. Multivariate analyses of RFS for TPBCs from the FUSCC cohort using Cox proportional hazards models.

	RFS	
Variables	HR (95% CI)	*P*
N category		
N0	Reference	–
N1-3	3.56 (1.23-10.30)	0.019
T category		
T1	Reference	–
T2-4	2.80 (1.06-7.44)	0.038
Subgroup		
non-luminal A-like	Reference	–
luminal A-like	0.33 (0.11-0.97)	0.045
Grade		
I-II	Reference	–
> II	1.91 (0.88-4.13)	0.100
Trastuzumab		
No	Reference	–
Yes	0.45 (0.21-0.97)	0.042
Radiotherapy		
No	Reference	–
Yes	1.24 (0.55-2.82)	0.601

Abbreviations: RFS: relapse-free survival; TPBC: triple-positive breast cancer; FUSCC: Fudan University Shanghai Cancer Center.

Supplementary Figures:

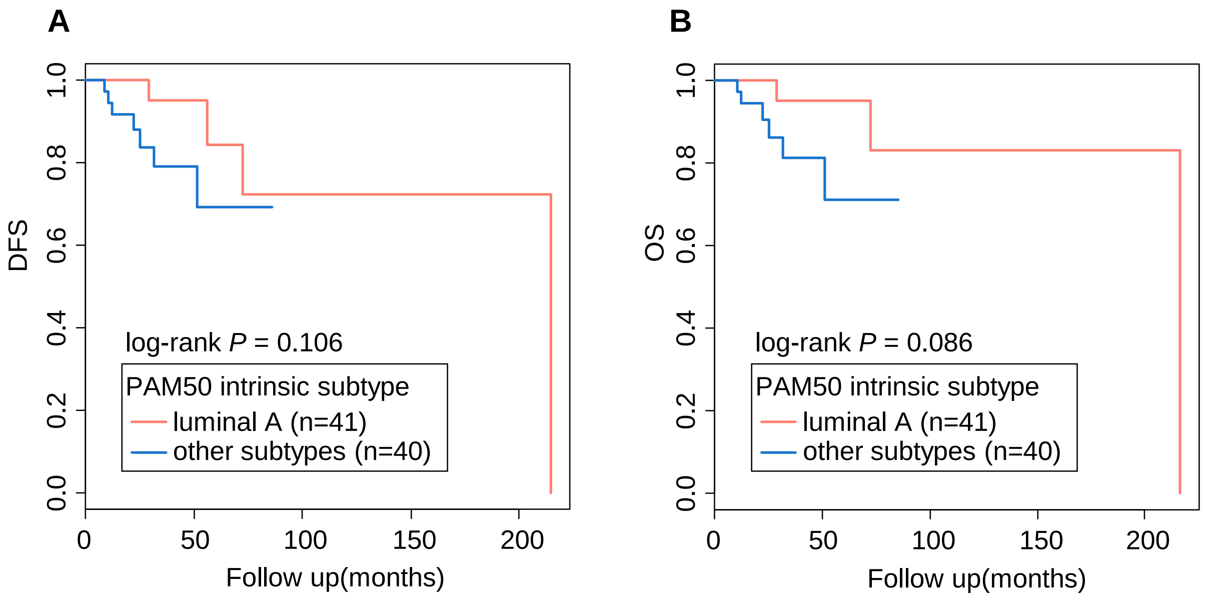

Figure S1. (A) DFS and (B) OS of TPBCs in the TCGA dataset.

P values are calculated using the log-rank test.

Abbreviations: TPBC: triple-positive breast cancer; DFS: disease-free survival; OS: overall survival.

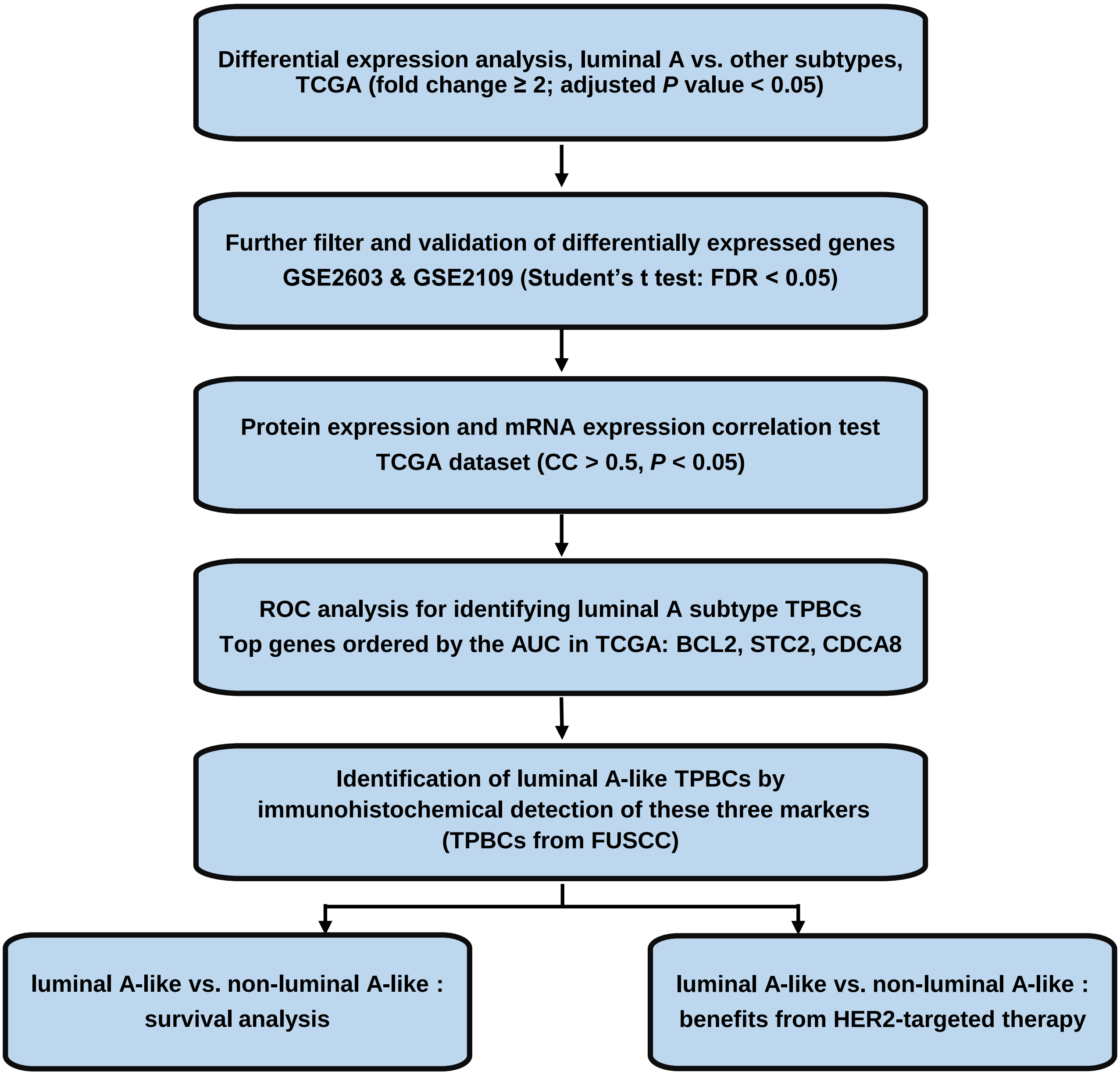

Figure S2. Workflow for the selection of genes that can be used to identify the luminal A subgroup of TPBCs.

Abbreviations: TPBC: triple-positive breast cancer; CC: correlation coefficient; ROC: receiver operating characteristic; AUC: area under the curve; FUSCC: Fudan University Shanghai Cancer Center.

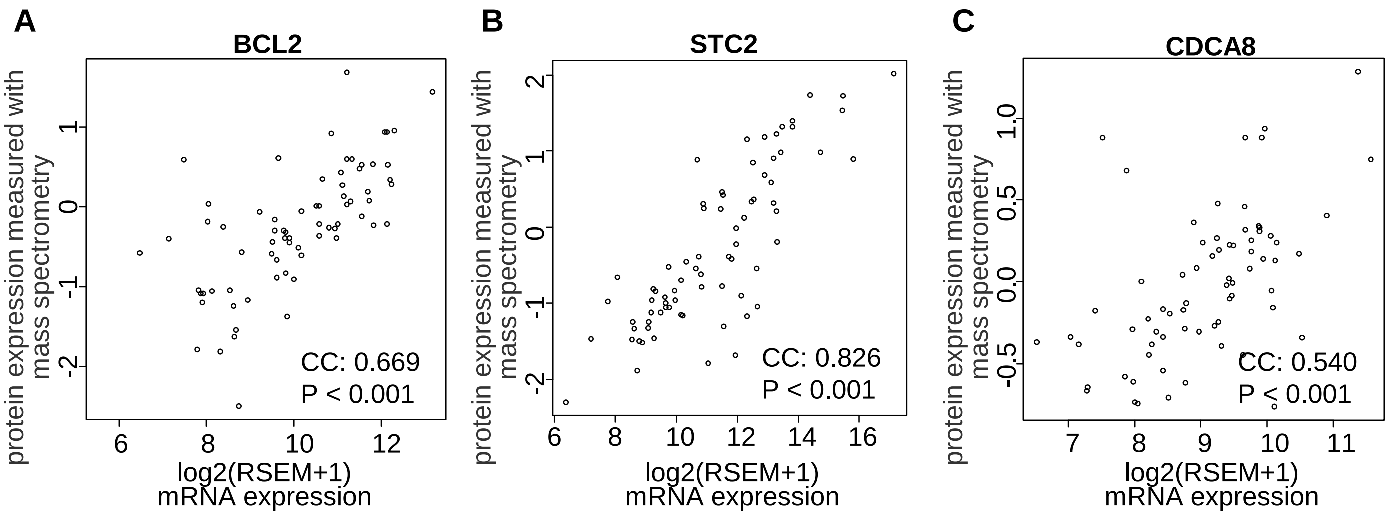

Figure S3. Correlation between the mRNA and protein expression of (A) BCL2, (B) STC2 and (C) CDCA8 in the TCGA dataset.

Correlation coefficients and *P* values are calculated using the Pearson correlation test.

Abbreviations: CC: correlation coefficient; RSEM, RNA-Seq by Expectation-Maximization.

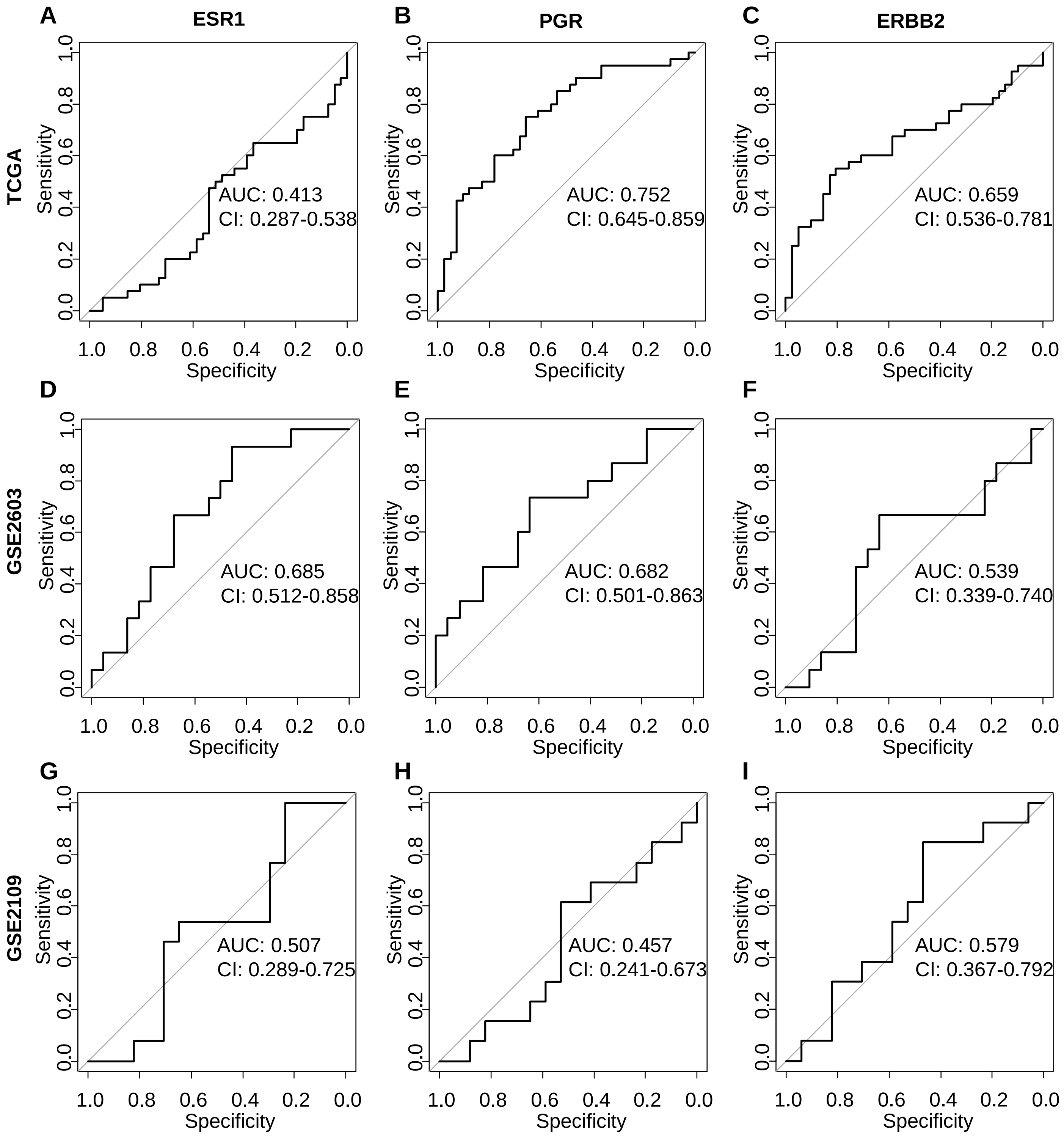

Figure S4. ROC curves of using the mRNA expression of ESR1, PGR or ERBB2 to identify luminal A subtype TPBCs in the (A-C) TCGA, (D-F) GSE2603 and (G-I) GSE2109 datasets.

Abbreviations: TPBC: triple-positive breast cancer; ROC: receiver operating characteristic; AUC: area under the curve; CI: confidence interval.

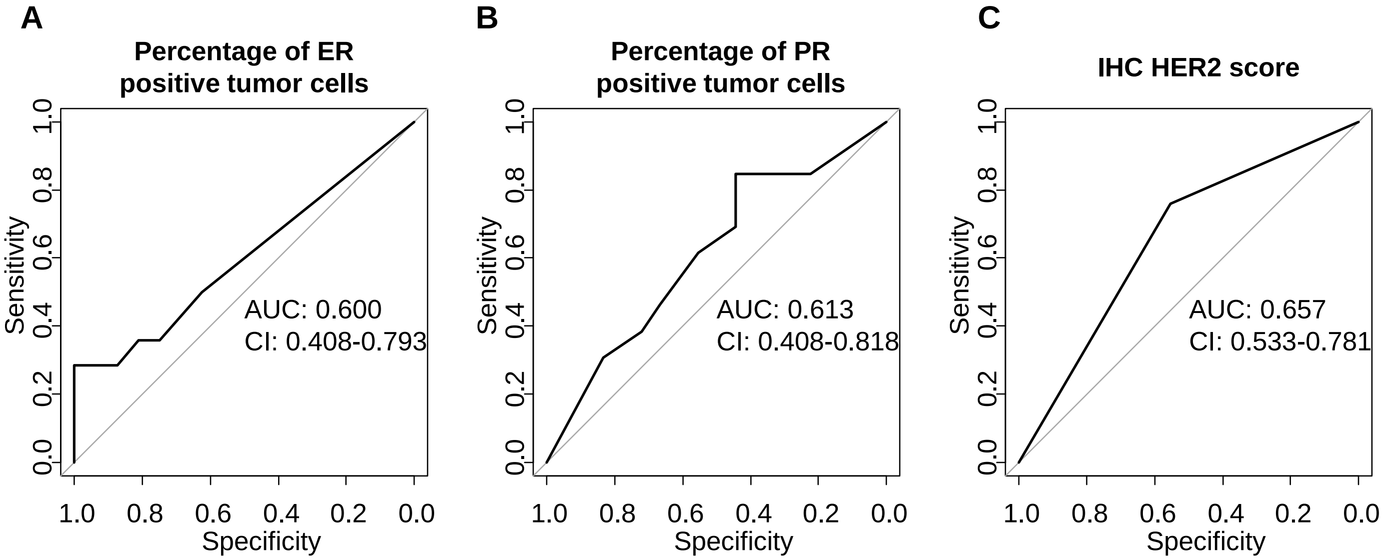

Figure S5. ROC curves of using the protein expression of (A) ER, (B) PR or (C) HER2 detected by immunohistochemistry to identify luminal A subtype TPBCs in the TCGA dataset.

Abbreviations: ER: estrogen receptor; PR: progesterone receptor; HER2: human epidermal growth factor receptor 2; TPBC: triple-positive breast cancer; ROC: receiver operating characteristic; AUC: area under the curve; CI: confidence interval.

A

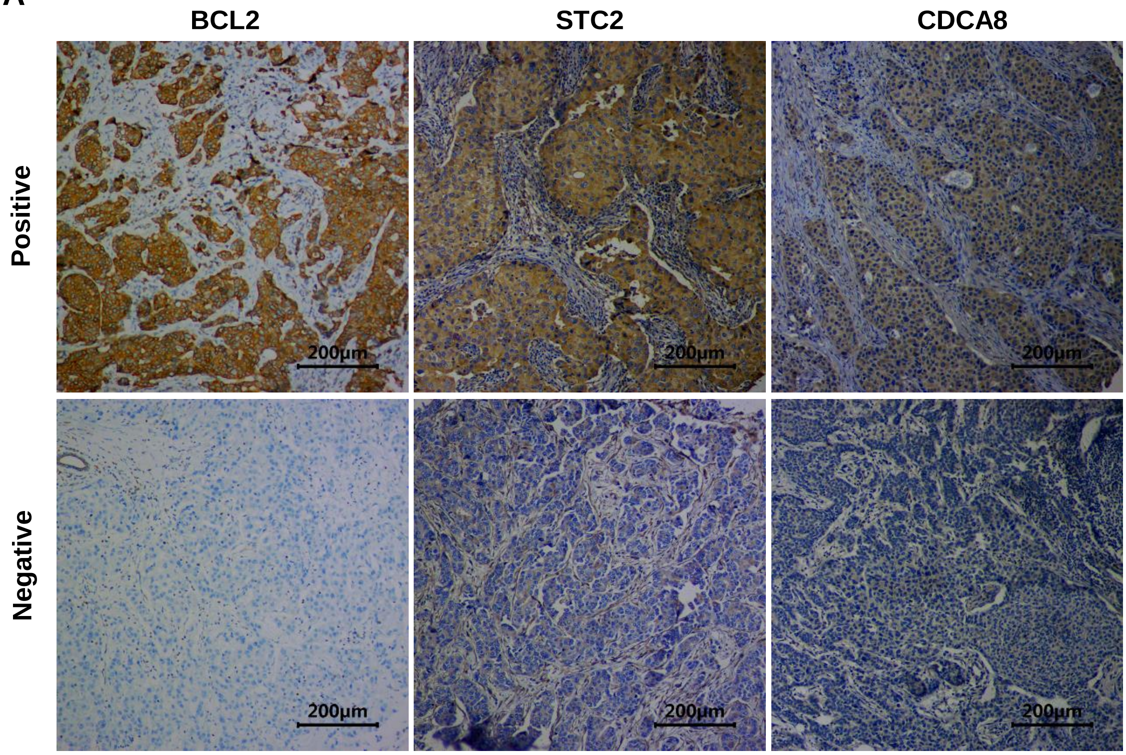

B

No. patients	BCL2	STC2	CDCA8
Positive	64	72	51
Negative	107	99	120

Figure S6. Immunohistochemical staining results of BCL2, STC2 and CDCA8.

(A) Representative images of BCL2, STC2 and CDCA8 immunohistochemical staining (*100).

(B) A summary of immunohistochemical staining results of BCL2, STC2 and CDCA8.

Abbreviations: TPBC: triple-positive breast cancer; FUSCC: Fudan University Shanghai Cancer Center.

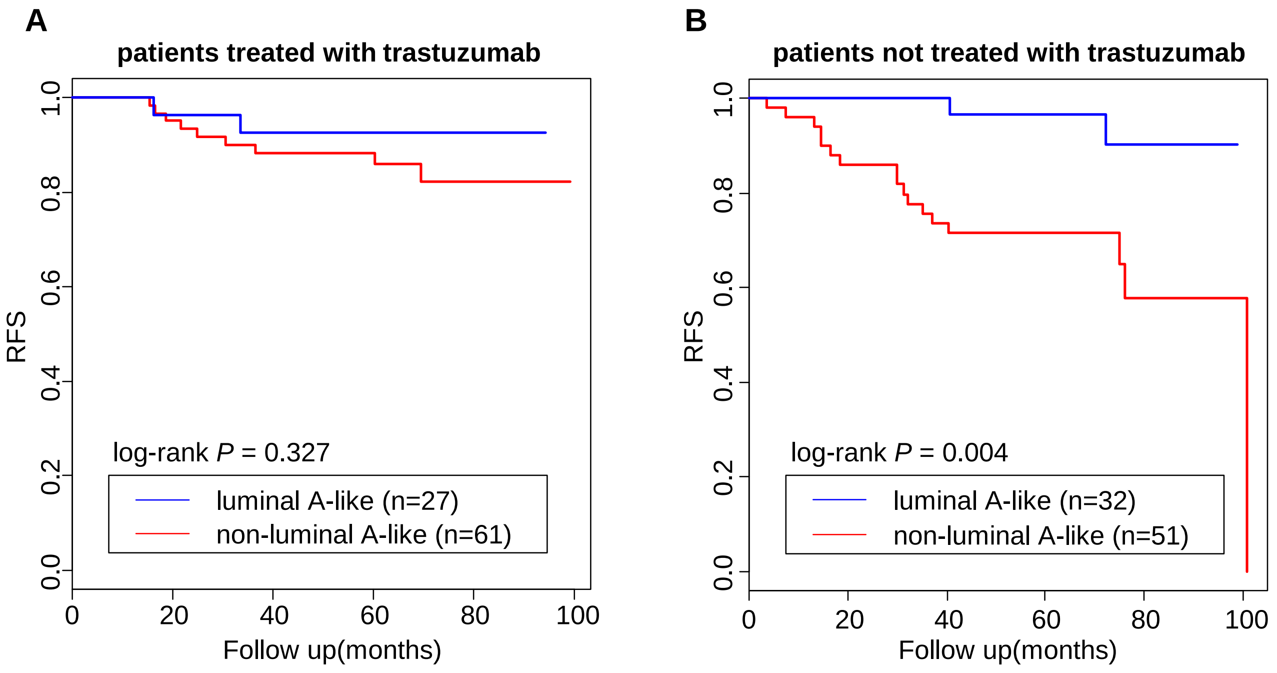

Figure S7. Comparison of relapse-free survival between the luminal A-like subgroup and the non-luminal A-like subgroup (A) in TPBC patients treated with trastuzumab and (B) in TPBC patients not treated with trastuzumab.

P values are calculated using the log-rank test.

Abbreviations: TPBC: triple-positive breast cancer; RFS: relapse-free survival.
